# Multiphysics Simulation in Drug Development and Delivery

**DOI:** 10.1007/s11095-022-03330-x

**Published:** 2022-07-07

**Authors:** Wenbo Zhan, Chi-Hwa Wang

**Affiliations:** 1 School of Engineering, King’s College, University of Aberdeen, Aberdeen, AB24 3UE UK; 2 Department of Chemical and Biomolecular Engineering, National University of Singapore, 4 Engineering Drive 4, Singapore, 117585 Singapore

Over the years, pharmaceutical research has made enormous contributions to human health care in preventing and treating diseases. In addition to the discovery of therapeutic compounds, it has also facilitated the development of various drug delivery systems and delivery methods. Despite these advances, the clinical efficacy remains to be improved, mainly due to the inherent physiological barriers and complex clinical situations. Disappointing success rates in drug development place high demands on bridging the gap between laboratory drug design and clinical practice to achieve precise, effective treatments.

In recent years, multiphysics simulation as an emerging technology has revolutionised drug development and delivery remarkably. Numerous models ranging from the macroscale to molecular scale have been applied to describe human in vivo environments and predict drug behaviours. According to the specific process, these models are established based on different principles, such as pharmacokinetics, pharmacodynamics, fluid mechanics, tissue mechanics, mass transport, bioheat transfer and biochemical reaction. This enables multiphysics simulation to integrate information from different stages of drug development, examine multiple interlinked delivery processes, and identify opportunities to maximise delivery outcomes and treatment effectiveness. Multiphysics simulation can not only reveal the mechanisms of drug delivery, but also provide a reference for formulating drug development guidelines.

## Highlights of the Special Issue

This special issue is commissioned to capture the state-of-art research efforts on multiphysics simulation in the areas of drug development and drug delivery, and to show their potential impacts on clinical care. It is composed of two expert reviews and ten original research articles.

Han and Ozcelikkale et al. [1] thoroughly reviewed the current efforts to model drug transport phenomena across scales and provided a critical analysis of remaining challenges. Focusing on drug delivery to the eye, Bhandari [2] contributed a comprehensive update on the modelling approaches for understanding fluid flow and mass transport in different ocular domains. The contributions of modelling studies to the existing treatments were also covered.

Li and Stinchcomb et al. [3] combined experiments and bottom-up simulations to explore how formulation factors determine drug transport kinetics across skin layers. Their study demonstrated the dominant role of diffusion, and more importantly, revealed its relationship with the water content and environmental temperature. Anissimov and co-workers [4] developed a microscale model to consider the superficial subpapillary dermal plexus and the effects of its size, depth, vessel density and blood flow on drug concentration. Model validation studies further denoted the superiority of this model in terms of predictive accuracy compared to previous ones.

Wang’s team [5] optimised salt compositions in methyl cellulose hydrogels for burn wound dressings. They employed a computational fluid dynamics model to examine the correlations between the structure of the printed hydrogel and the printing parameters. McGinty et al. [6] quantified the influence of fluid flow on the drug release rate of drug-filled implants with different release strategies (a porous pin with pores in μm and a pin drilled with orifices in mm), and for each strategy a suitable release model was identified. Xu and co-workers [7] tested the targeted thrombolysis using activated tissue plasminogen nanovesicle (tPA-NV) under 16 therapeutic scenarios. Their study showed that tPA-NV was superior to conventional therapy in reducing the dose, rapidly recanalizing the lumen, and reducing the risk of bleeding complications.

In this recent work by Wang et al. [8], a mechanistic model was set up and extrapolated to the human scale to evaluate the potential of miRNA-22 nanotherapy in the treatment of triple-negative breast cancer (TNBC). Their studies showed the importance of combining with immune checkpoint inhibitors and elucidated the drug synergy between miRNA-22 and the current course of TNBC treatment. Soltani et al. [9] drew their expertise in predicting the response of thermosensitive liposome-mediated drug delivery to magneto-hyperthermia duration. Their study suggested that optimal delivery results could be achieved when heating started after bolus injection until the drug concentration reached its peak in the tumour extracellular space. Yuan and Dini et al. [10] established a particle tracking model to capture the trajectories of nanoparticles in the brain white matter. Their study showed that zeta potential rather than nanoparticle size played a more important role in determining the particle diffusivity, whereas this importance was less pronounced when the value was less negative than -10 mV. Zhan and co-worker [11] investigated the impact of tumour tissue permeability on convection-enhanced delivery based on a 3D realistic brain tumour model. The hydraulic environment was more friendly for drug transport in permeable tumours. Tissue permeability and blood pressure were more critical for delivery outcomes than brain ventricular permeability.

Perivilli and colleagues [12] conducted a design of experiments-analysis to evaluate the individual and cross-influence of multiple factors on the hydrodynamics in paddle apparatus. The impeller offset was found to be the dominating parameter that can affect overall fluid flow. In contrast, the rest parameters including the distance between vessel and impeller bottom surface, vessel dimension and impeller rotating speed had limited impact, which was mainly restricted at locations near the vessel wall.

## Remarks

Multiphysics simulation has been widely applied in drug development and drug delivery. In addition to the applications discussed in this collection, it has shed light on a variety of delivery means and disease treatments, drug formulation design, and drug fabrication and testing. As an open platform, a mathematical model allows being tailored to couple multiple physical, chemical and biological processes that are involved in a single drug delivery and/or development activity. This opens a cost-effective avenue for exploring the underlying mechanisms and enables utilising realistic patient information, which will facilitate the development of personalised, precise treatment.

Models are developed at different scales. Dividing the entire biological system like the human body into multiple compartments, pharmacokinetics (PK) models can fast predict the time course of drug concentration and analyse the drug-drug interactions, drug-tissue interactions and drug exchange between compartments. The combination with pharmacodynamics (PD) models further enable describing the response of the studied system to drugs. Given these advantages, PK/PD models have been seen significant adoption to optimise dose and regimen [13], develop effective drugs [14], scale laboratory experiments to clinical trials [15], and explore and examine the physiological barriers in drug delivery [16], particularly the delivery to the central nervous system [17, 18]. Unlike PK/PD models, transport-based models are able to accommodate the realistic geometry of the entire tissue or tissue microstructure for outputting both the temporal drug concentration and spatial distribution. This feature makes the transport-based model suitable for considering the heterogeneous and/or anisotropic intra-tissue environments [19]. However, studies using these models usually concentrate on a single tissue, while the rest of the body would be ignored or simplified. A cross-scale model coupling the transport-based model with PK/PD model would help to overcome this limitation. Moreover, molecular dynamics is now attracting more attention in drug development. As an assumption-free approach, it provides an effective means to observe the interactions of drug molecules and biomolecules [20]. However, its computational domain and simulation time window are usually limited. Importantly, the predictive ability and application of the models must be validated in advance to ensure quality [21].

It is worth noting that as model complexity increases, the demand for computational power and time would raise dramatically, which would become a bottleneck of multiphysics simulation. Recent advances in machine learning [22, 23] could provide a potential solution for rapidly solving the governing equations, particularly the cross-linked partial differential equations.

We expect that this collection will highlights recent progress in multiphysics simulation for a broad spectrum of applications in drug development and drug delivery, and accelerate the translations to pharmaceutical and clinical practice. Finally, we would like to thank all the authors, reviewers and journal editors for their invaluable efforts and support.

## Declaration

The authors have declared that no competing interests exist.
